# The mechanism for enhanced oxidation degradation of dioxin-like PCBs (PCB-77) in the atmosphere by the solvation effect

**DOI:** 10.1186/s13065-017-0291-3

**Published:** 2017-07-11

**Authors:** Mei-Ling Xin, Jia-Wen Yang, Yu Li

**Affiliations:** 1 College of Environmental Science and Engineering, North China Electric Power University, No. 2, Beinong Road, Beijing, 102206 China; 2 The Moe Key Laboratory of Resources and Environmental Systems Optimization, North China Electric Power University, Beijing, 102206 China

**Keywords:** PCB-77, Density functional theory, Atmospheric oxidant, Degradation pathway, Solvation effect

## Abstract

The reaction pathways of PCB-77 in the atmosphere with ·OH, O_2_, NO_*x*_, and ^1^O_2_ were inferred based on density functional theory calculations with the 6-31G* basis set. The structures the reactants, transition states, intermediates, and products were optimized. The energy barriers and reaction heats were obtained to determine the energetically favorable reaction pathways. To study the solvation effect, the energy barriers and reaction rates for PCB-77 with different polar and nonpolar solvents (cyclohexane, benzene, carbon tetrachloride, chloroform, acetone, dichloromethane, ethanol, methanol, acetonitrile, dimethylsulfoxide, and water) were calculated. The results showed that ·OH preferentially added to the C5 atom of PCB-77, which has no Cl atom substituent, to generate the intermediate IM5. This intermediate subsequently reacted with O_2_ via pathway A to generate IM5a, with an energy barrier of 7.27 kcal/mol and total reaction rate of 8.45 × 10^−8^ cm^3^/molecule s. Pathway B involved direct dehydrogenation of IM5 to produce the OH-PCBs intermediate IM5b, with an energy barrier of 28.49 kcal/mol and total reaction rate of 1.15 × 10^−5^ cm^3^/molecule s. The most likely degradation pathway of PCB-77 in the atmosphere is pathway A to produce IM5a. The solvation effect results showed that cyclohexane, carbon tetrachloride, and benzene could reduce the reaction energy barrier of pathway A. Among these solvents, the solvation effect of benzene was the largest, and could reduce the total reaction energy barrier by 25%. Cyclohexane, carbon tetrachloride, benzene, dichloromethane, acetone, and ethanol could increase the total reaction rate of pathway A. The increase in the reaction rate of pathway A with benzene was 8%. The effect of solvents on oxidative degradation of PCB-77 in the atmosphere is important.

**Graphical abstract:**
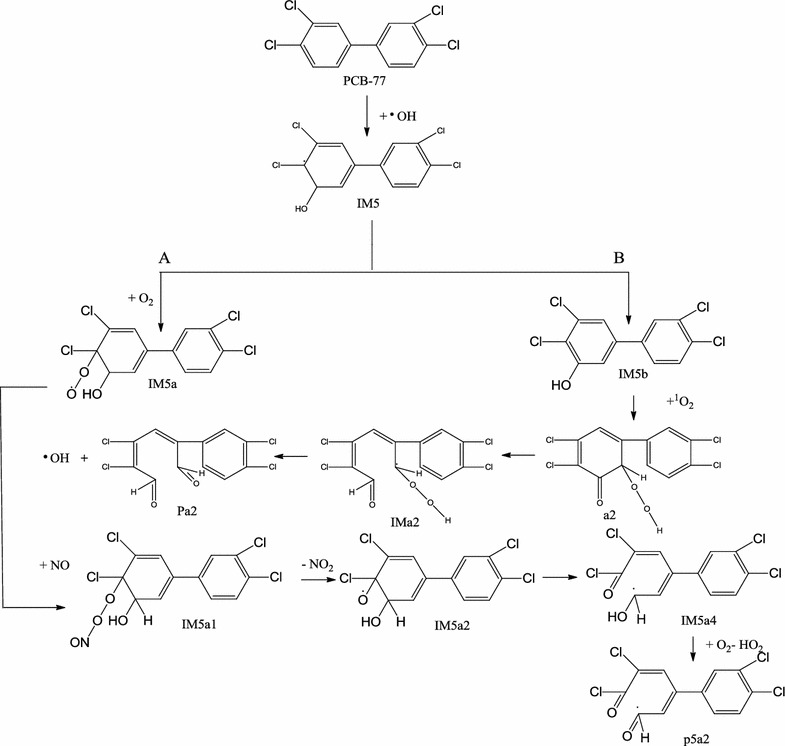
The reaction pathways of PCB-77 in the atmosphere with •OH, O2, NOx, and 1O2 were inferred based on density functional theory calculations with the 6-31G* basis set. Different polar and nonpolar solvents: cyclohexane, benzene, carbon tetrachloride, chloroform, acetone, dichloromethane, ethanol, methanol, acetonitrile, dimethylsulfoxide, and water were selected to study the solvation effect on the favorable reaction pathways. The investigated results showed what kind of pathway was most likely to occur and the solvent effect on the reaction pathway

The reaction pathways of PCB-77 in the atmosphere with •OH, O2, NOx, and 1O2 were inferred based on density functional theory calculations with the 6-31G* basis set. Different polar and nonpolar solvents: cyclohexane, benzene, carbon tetrachloride, chloroform, acetone, dichloromethane, ethanol, methanol, acetonitrile, dimethylsulfoxide, and water were selected to study the solvation effect on the favorable reaction pathways. The investigated results showed what kind of pathway was most likely to occur and the solvent effect on the reaction pathway

## Introduction

### The pollution influence of PCBs

Polychlorinated biphenyls (PCBs) have been widely used as flame retardants, dielectric and heat transfer fluids, and plasticizers in industrial and household products [1]. As typical chlorinated organic pollutants, large quantities of PCB byproducts are released into the environment causing serious pollution and adverse impacts on ecosystems [2, 3]. Because of their volatility, persistence, bioaccumulation, and high toxicity, they have been listed by the United Nations Environment Programme as one of 12 kinds of persistent organic pollutants that need to be controlled [4]. The physical and chemical properties of PCBs are very stable. Once PCBs come into the environment, they remain in the environment for a long time and are very difficult to degrade (half-life is about 40 years) [5]. PCBs are not degraded by hydrolysis or similar reactions in the environment at a marked rate, and only a small fraction of PCBs are converted by photolysis in the soil and by photolysis of sunlight and ultraviolet light. Contaminated water and soil are also difficult to recover and have high residual in the environment. Under the stress of PCBs, the whole community structure of soil bacteria will change, which leads to the change of soil ecological environment quality [6]. Because of its lipophilic and hydrophobic characteristics, its long-term accumulation and the high bioaccumulation of some isoforms and congeners, PCBs have strong accumulation in the organism and are gradually enriched through the food chain. If the water contains 0.01 μg/L PCBs, its accumulation in the fish can reach 20 × 10^4^ times of the concentration of water, and which in the body of birds and animals who eat fish will be higher. Some big fish in the sea and fierce birds in the air, such as sharks, seals and raptors, the concentration in their body can be 10^7^–10^8^ times higher than the surrounding environment [7]. The characteristics of the PCBs enable them to be migrated remotely and can be dispersed globally through the pathway of volatiles—atmospheric transport—sedimentation. From Antarctic penguins to the Arctic seals, they have been detected PCBs in their bodies. PCBs pollution has become a global problem. Related toxicological studies have shown that PCBs have an induced effect on lesions, or even concretization, of skin, liver, gastrointestinal system, nervous system, reproductive system, and immune system. Some homologues affect the reproduction of mammals and birds, and are potentially carcinogenic to human health. They are prone to accumulate in adipose tissue, causing brain, skin and visceral diseases and affect the nervous, reproductive and immune systems [8].

### The industrial recycling options of PCBs

The PCBs are the platform upon which microelectronic components such as semiconductor chips and capacitors are mounted. PCBs provide the electrical interconnections between components and are found in virtually all electrical and electronic equipment. Every year, 20–50 million tons of waste electrical and electronic equipment (WEEE) are generated worldwide, which could bring large amount of waste PCBs. Recycling of waste PCBs is an important subject not only from the treatment for waste but also from the recovery of precious metal, The typical metals in PCBs consist of copper, iron, tin, nickel, lead, zinc, silver, gold, and palladium [9]. Due to its complex composition, PCBs recycling requires a multidisciplinary approach intended to separate fibers, metals and plastic fractions and reduce environmental pollution. Recycling process for waste PCBs includes three processes which is pretreatment, physical recycling, and chemical recycling. PCBs recycling generally start from the pretreatment stage, which include disassembly of the reusable and toxic parts and then PCBs are treated using physical recycling or chemical recycling process [10]. In general, the recycling methods of waste PCBs can be summarized as physical recycling methods and chemical recycling methods. Physical processing for the separating the metal fraction and non-metal fraction from waste PCBs includes shape separation, magnetic separation, electric conductivity-based separation, density-based separation and corona electrostatic separation. The chemical recycling methods include pyrolysis, gasification and combustion. Metal fraction can be treated by pyrometallurgical, hydrometallurgical or biotechnological process [11].

Industrial recovery of PCBs is indeed an environmentally friendly technology and 85% of the PCBs wastes produced by industry are to be recycled [12]. Furthermore, Large amount of the PCBs wastes were untreated wastes and exposed to wind and rain, easy to evaporate into the atmosphere or deposited into the soil, which can pose a serious threat to local water, air environment and human health, and lead to PCBs are not easy to be recycled.

### The atmospheric degradation research status of PCBs

Pathways for transformation and removal of PCBs in the environment are a hot topic. Because PCBs degrade very slowly, they are now ubiquitous in air, water, soil, sediment, and biota. During transportation, PCBs can be removed and transformed through photolysis, wet and dry deposition, and chemical reactions with ·OH, NO_3_, Cl radicals, and O_3_. The radical ·OH is considered to be the main initiator for the removal of volatile organic compounds in the atmosphere [13–15]. The reaction with ·OH is considered the dominant removal pathway of PCBs from the atmosphere [16]. Rate constants for the gas phase reactions of 14 PCB congeners with ·OH have been measured from 323 to 363 K, the experimental data suggested that the more highly chlorinated PCBs would become progressively less reactive with OH radicals, which would increase their lifetimes compared to lower chlorinated PCBs [17]. Using the MPWB1K functional, Sun et al. [18] investigated the degradation process of PCB-47 with oxygen and nitrogen oxides, and found that the main degradation product of PCB-47 was glyoxal, the reaction rate with ·OH was 1.27 × 10^−12^ cm^3^/molecule s, and the half-life was 9.1 days. Lee et al. [19] studied the mechanisms for the formation of polychlorinated dibenzodioxin–OH adducts using density functional theory (DFT), and found that carbon atoms connected to oxygen atoms were the main sites of ·OH addition. Altarawneh et al. studied the atmospheric degradation of polychlorinated dibenzofurans initiated by ·OH addition and found a reaction rate of 2.70 × 10^−11^ cm^3^/molecule s. They suggested that the ·OH adduct immediately reacted with O_2_ to generate polychlorinated dibenzofuran–OH–O_2_ adducts [20]. Because of the reactivity of ·OH, most studies on the reactions of persistent organic pollutants with free radicals or reactive molecules in the atmosphere have focused on ·OH [21]. Because of the complexity of environmental conditions, it is difficult to use computational and theoretical chemistry to accurately evaluate environmental effects, and particularly the effect of solvents. More complete reaction mechanisms for oxidative degradation induced by ·OH, and studies of the effects of different solvents on the reaction mechanisms are required.

### Research content

Quantitative structure activity relationship studies have shown that PCBs with meta- and para-chlorine substituents are the most similar to dioxins among the PCBs. Toxicological studies have shown that PCBs that are not coplanar or ortho-substituted, such as the 14 dioxin like PCBs (congener numbers 77, 81, 105, 114, 118, 123, 126, 156, 157, 169, 170, 180, and 189) have high reactivity and toxicity [22]. In the present study, PCB-77 (3,3′,4,4′-Tetrachlorobiphenyl), which has two meta- and two para-chlorine substituents, was selected for study by quantum chemistry calculations using DFT. The results were used to predict the degradation reactions of PCBs with the common oxidants ·OH, O_2_, NO_*x*_, and ^1^O_2_. The intermediates and transition states in the reactions were predicted, and the rate constants were calculated by transition state theory (TST). The effects of different solvents on the reaction pathways were studied based on the solvation effect.

## Computational methods

The Gaussian 09 program [23] was used to perform all calculations. The degradation reactions of PCB-77 were optimized for all reactants, transition states, intermediates, and products. The optimal structure was obtained using DFT at the B3LYP/6-31G*level. This method has yielded satisfying results in previous research [24, 25]. The vibrational frequencies were calculated at the same level, and showed that the transition state has only one imaginary frequency, and the intermediate for each transition state has no imaginary frequency. The intrinsic reaction coordinate was calculated to identify the connections between reactants, transition states, and products. To obtain more accurate Gibbs free energies, the basis set of 6-311G(2df, p) was used with the same method to calculate the single point energies based on the optimal material configuration, taking into consideration the zero point energy correction. The reaction rates at 298 K and standard atmospheric pressure were calculated using transition state theory (TST), taking into consideration the tunneling effect, with KiSTheIP software [26]. The polarizable continuum model (PCM) was used to optimize the reaction parameters of the intermediates and transition states of PCB-77 degradation, and analyze the effect of solvation. PCM is widely used in studies of the solvation effect as the classical quantum chemical calculation method [27]. Many studies show that the results of PCM can be directly compared with the outcome of the experimental measurements,and the results are in good agreement with the experimental values [28–30]. To eliminate systematic error between the theoretical and experimental values, the calculations were multiplied by a calibration factor of 0.960 [31].

## Results and discussion

### The reaction of PCB-77 with ·OH

**Fig. 1:**
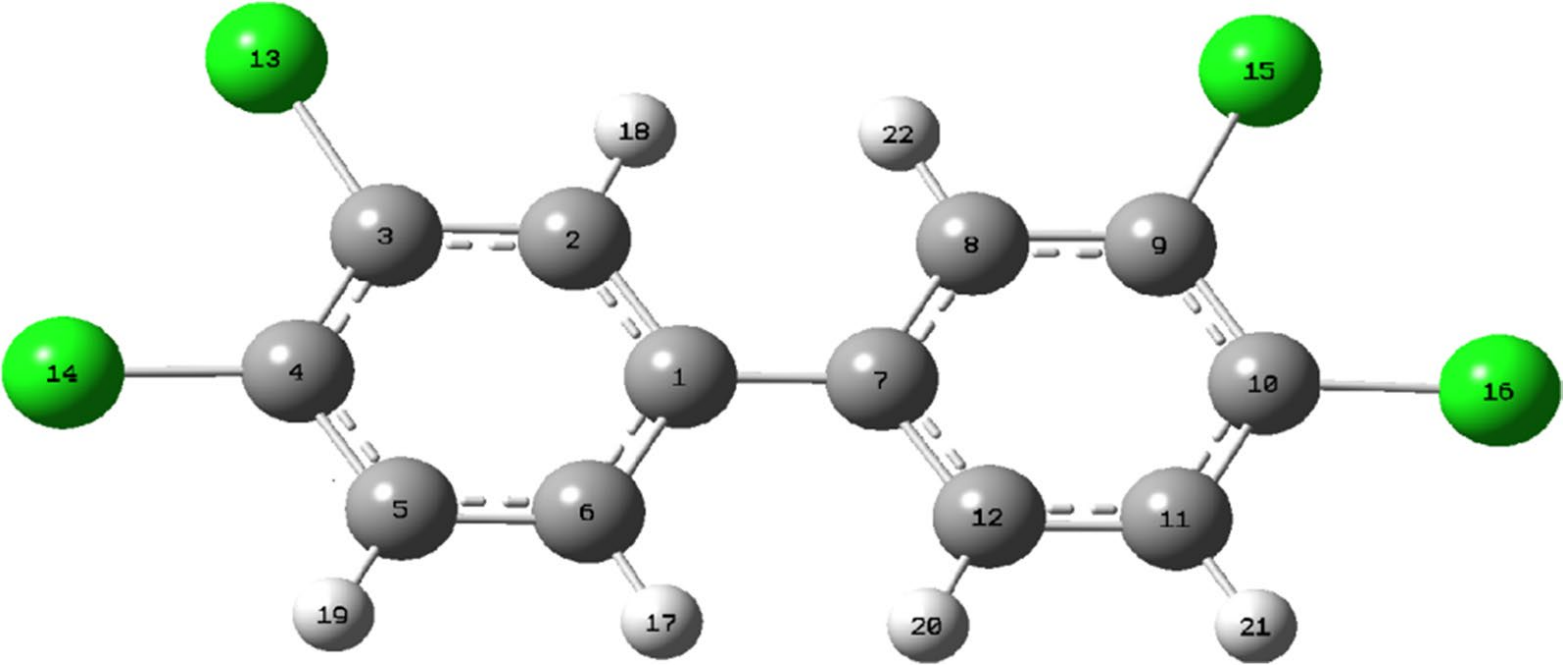
Optimized structure of PCB-77

**Fig. 2:**
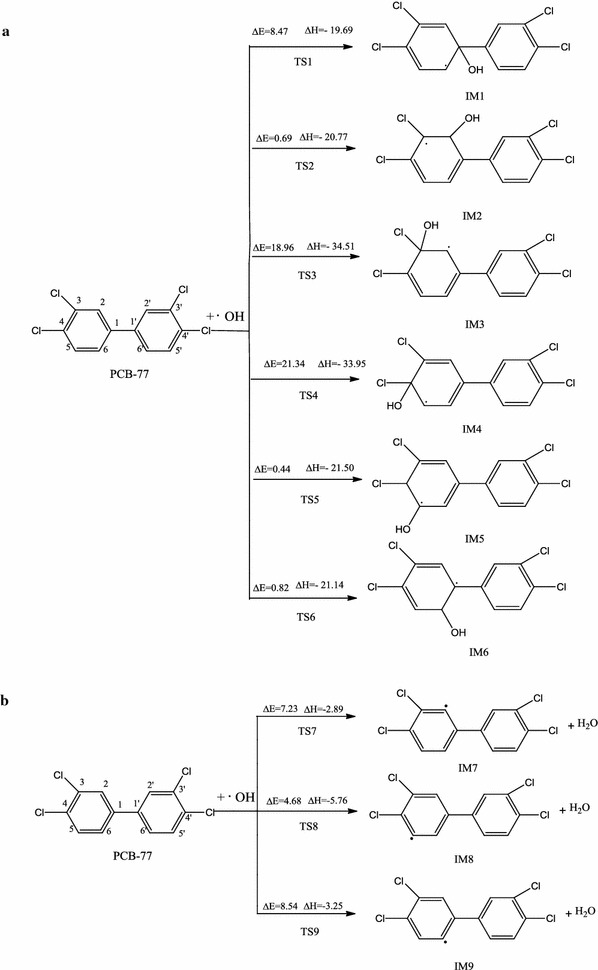
The reaction pathway of PCB-77 with ·OH (**a** for the addition reaction rath, **b** for the H extraction reactionpath) [∆E (kcal/mol): energy barrier, ∆H (kcal/mol): reaction heats]

**Fig. 3:**
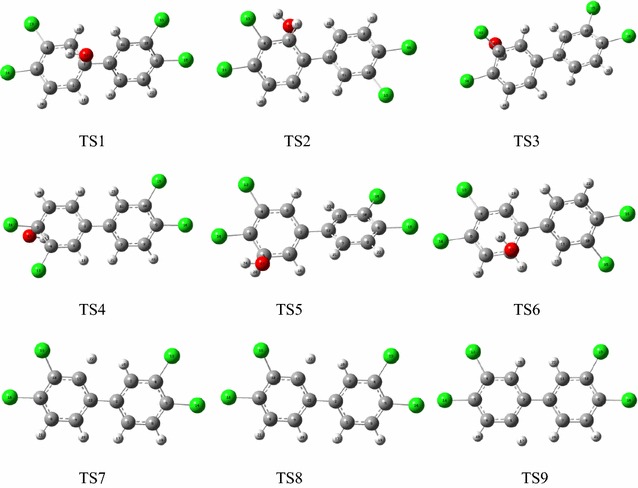
The optimization structure of transition state of PCB-77 with ·OH

Because of the large proportion of oxygen in the atmosphere, ·OH is considered to be the most important atmospheric oxidant. This ·OH can react with environmental pollutants via either ·OH addition or H abstraction. The possible pathways for the reactions of PCB-77 with ·OH radicals are shown in Figs. 2 and 3. PCB-77 (Fig. 1) shows C1 symmetry, and contains six different carbon atoms that could be sites for ·OH addition. Among the six transition states for these carbon atoms (TS1–TS6, Fig. 2a), TS3 and TS4 present the highest energy barriers to ·OH addition. This can be attributed to the steric hindrance of the chlorine atom, which can block ·OH addition to C3 and C4. The energy barrier for TS1 is much higher than those for TS2, TS5, and TS6, mainly because of the position of C1 and steric hindrance from the benzene ring. The energy barriers for ·OH addition to C2, C5, and C6 are relatively low, and ·OH addition is likely to occur at these sites. Among the possible pathways, addition of ·OH to the C5 atom to form the intermediate IM5 is the most favorable pathway with the lowest energy barrier (0.439 kcal/mol), and is exothermic (21.52 kcal/mol). Three direct H abstraction pathways were identified (Fig. 2b). These were H atom abstraction from the C2–H, C5–H, and C6–H bonds. Formation of IM8 is the most favorable pathway with the lowest energy barrier (4.68 kcal/mol), and is exothermic (5.76 kcal/mol). These results confirm that the C5 atom is more reactive than the other carbon atoms. Consequently, IM5 and IM8 were selected for further investigation. When the ·OH addition and H abstraction were compared, the H abstraction reaction had a higher energy barrier than the addition reaction, and was less exothermic. PCB-77 preferentially occurred addiction reaction with OH, which is consistent with the results of Chen et al [32–34]. The Optimization structure of transition state (TS1–TS9) as shown in Fig. 3.

### Continuous addition reaction pathway of IM5 and O_2_

**Fig. 4:**
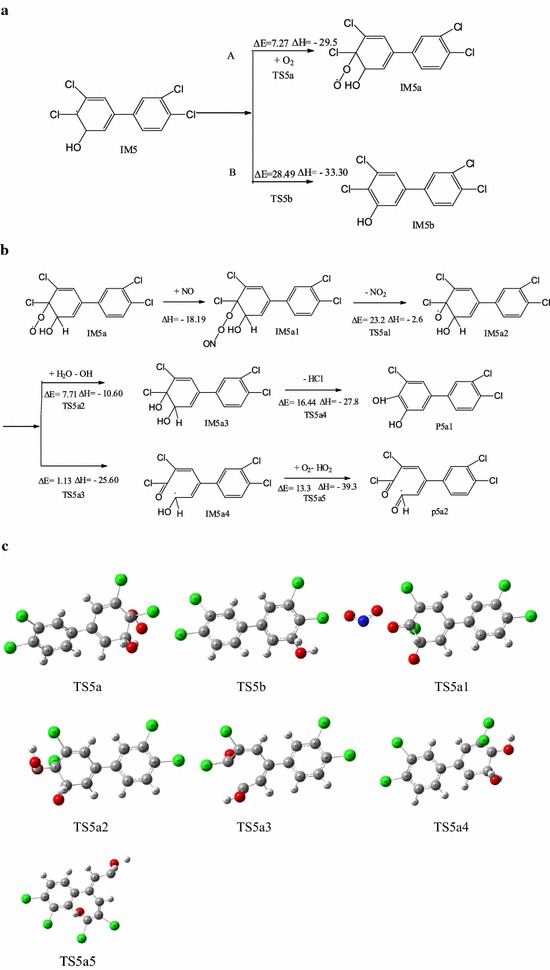
Two stage reaction pathway of intermediate IM5 (**a**), reaction pathway of intermediate IM5a with NO (**b**), and the transition state structure of IM5a with O_2_/NO (**c**). [∆E (kcal/mol): energy barrier, ∆H (kcal/mol): reaction heats]

The addition reaction of PCB-77 and ·OH to produce the reactive intermediate IM5 can be followed by two pathways (Fig. 4a). Path A involves direct addition of O_2_ to IM5 to generate IM5a. Path B involves H abstraction of IM5 to generate OH-PCBs (IM5b). The energy barrier for O_2_ addition was 7.27 kcal/mol, and the reaction was exothermic (29.5 kcal/mol). The energy barrier for direct H abstraction was 28.49 kcal/mol, and the reaction was exothermic (33.30 kcal/mol). Therefore, under these conditions, intermediate IM5 easily reacts with O_2_ to form IM5a. IM5a contains peroxy radicals, is strongly reactive, and will react with other substances in the air. Because NO is a free radical with a single electron, it can participate in many free radical reactions [35]. The reaction of IM5a and NO was evaluated (Fig. 4b). The intermediate IM5a1 was obtained via direct NO addition to the peroxy radical, and this reaction was barrierless and exothermic (18.19 kcal/mol). Single molecule decomposition of IM5a1 generates IM5a2 and NO_2_, with an energy barrier of 23.20 kcal/mol in an exothermic reaction (2.60 kcal/mol). IM5a2 can generate products P5a1 and P5a2 by bimolecular reaction and self-decomposition. IM5a2 can generate diol product P5a1 and ·OH, with an energy barrier of 7.71 kcal/mol in an exothermic reaction (10.60 kcal/mol). The newly produced ·OH initiates a new round of PCB degradation. This phenomenon has been found in the atmospheric oxidation of polychlorinated dibenzodioxins/furans in the presence of water vapor [36]. The ring-opening reaction of IM5a2 to produce P5a2 occurs by cleavage of the C4–C5 bond, and the oxygen free radicals are converted to double bonds. The energy barrier for this reaction is 1.13 kcal/mol, and it is exothermic (25.60 kcal/mol). Comparison of the two pathways indicated that IM5a2 was more prone to self-decomposition. The Optimization structure of transition state (TS5a–TS5a5) as shown in Fig. 4c.

### Addition reaction pathway of IM8

**Fig. 5:**
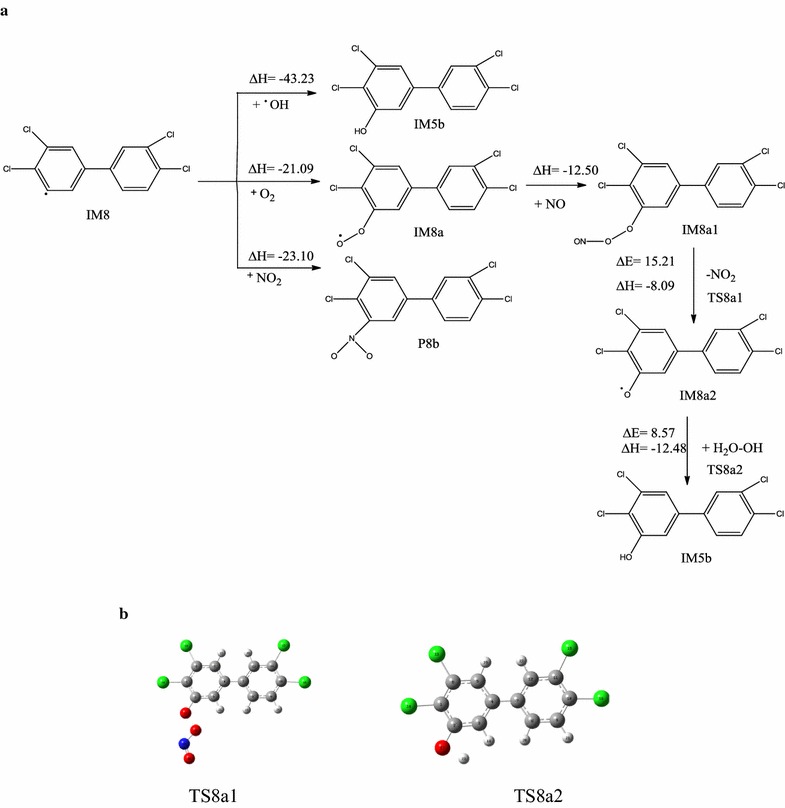
Addition reaction pathway of intermediate IM8 with ·OH, O_2_, NO_2_ (**a**) and the transition state structure of IM8a with NO (**b**). [∆E (kcal/mol): energy barrier, ∆H (kcal/mol): reaction heats]

IM8 is strongly reactive with a single electron, and can react with larger molecules or radicals in the atmosphere. A possible reaction pathway is shown in Fig. 5a. The addition reactions of IM8 with ·OH, O_2_, and NO_2_ are barrierless and exothermic (43.23, 21.09, and 23.10 kcal/mol, respectively). The addition reaction of IM8 with ·OH also produces IM5b. The reaction of IM8 with O_2_ generates IM8a, which contains the dioxygen radical. The reaction pathway is similar to that for IM5a in the atmosphere, and finally generates IM5b. Addition of NO_2_ produces P8b. The Optimization structure of transition state (TS8a1, TS8a2) as shown in Fig. 5b.

### Addition pathway of intermediate IM5b with singlet oxygen (^1^O_2_)

Reactions of IM5 and IM8 in the atmosphere can generate IM5b, which is hydroxylated PCBs. OH-PCBs have been widely detected in the abiotic environment in air, rain, and snow. These compounds have potential estrogenic activity, thyroid effects in vitro and in vivo, and are toxic [37, 38]. ^1^O_2_ is an important reactive oxygen species. The electron configuration of its molecular orbital is different from ground state oxygen. ^1^O_2_ is a form of excited state molecular oxygen, and can easily react with unsaturated compounds because of its high activity. Studies have found that chlorophenol and ^1^O_2_ reactions are the main contributors to decomposition [39]. We deduced an addition reaction pathway of IM5b with ^1^O_2_. Generally, the following four principal types of oxygen addition reactions that produce aromatic and unsaturated compounds are recognized [40]: (1) 1,3-addition to a double bond connected to a hydrogen-carrying group to form allylic hydroperoxides; (2) 1,4-cycloaddition to a system containing at least two conjugated double bonds to form 1,4-peroxides; (3) [p2 + p2] 1,2-cycloaddition to an isolated double bond to form 1,2-peroxides; and (4) 1,4-addition to phenols and naphthols to form hydroperoxide ketones.

**Fig. 6:**
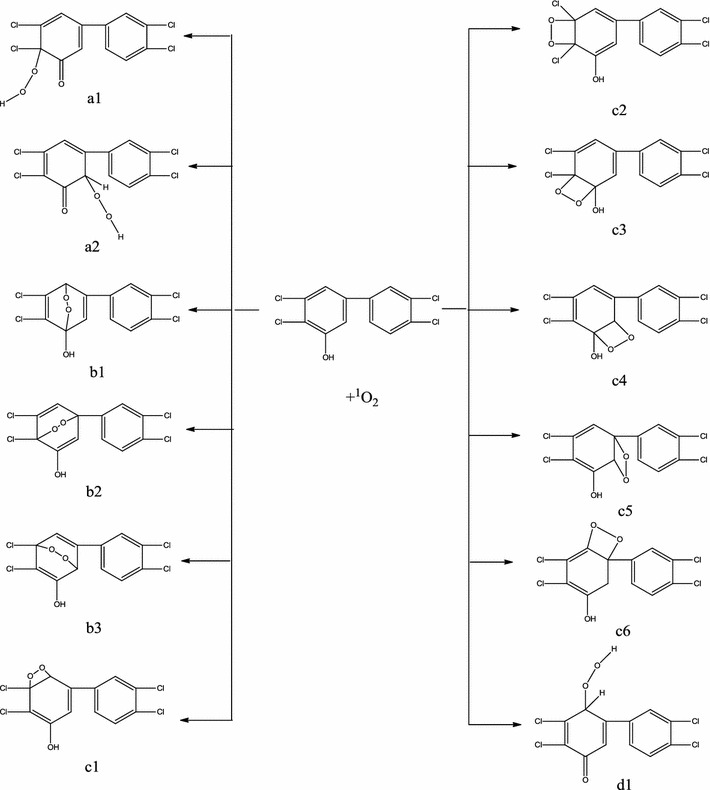
Twelve reaction pathways of IM5b with ^1^O_2_

Between IM5b and singlet oxygen, these four types of reactions would, in principle, occur in a similar way (Fig. 6). The optimal structures and thermodynamic properties of reactants, transition states, and products of the 12 reaction paths of IM5b with ^1^O_2_ were calculated. For each reaction pathway, the Gibbs free energy change (∆G) was calculated (Table 1). Reactions a1, a2, c1, c2, c3, c4, and d1 all had negative ∆G, and could be spontaneous. The ∆G of reactions b1, b2, b3, c5, and c6 were positive. For the reaction of 2, 4-chlorophenesic acid with ^1^O_2_, the ∆G of reaction pathways a and d were negative and those of reaction pathways b and c were positive [41]. In contrast to the results of Song et al. in this paper, the ∆G of pathways c1–c4 were negative. This could be because IM5b has meta- and para-chlorine substituents, whereas the 2,4-dichlorophenol studied by Song et al. has ortho- and para-chlorine substituents. The meta-chlorine atoms could change the benzene ring electron distribution and reduce the stability of the benzene ring. This could mean the addition reaction could occur, and ^1^O_2_ could add to the double bond of the benzene ring so that the reaction pathway of c1–c4 could be spontaneous. The reaction pathway of c5 and c6 cannot be spontaneous, because the C1 atom is attached to two benzene rings, and it is not easy for the carbon in this position to react because of conjugation. However, the thermodynamic study only suggests this is reaction possible, the reaction mechanism still needs to be confirmed by dynamics calculations. Next, we calculated the reaction energy barriers (Table 2) and determined the optimal pathway.

**Table 1:** Gibbs free energy change (∆G) of twelve reaction pathways unit: Kcal/mol

Reaction type a	∆G	Reaction type b	∆G	Reaction type c	∆G	Reaction type d	∆G
*a1*	*−3.3* *×* *10* ^*−2*^	b1	1.0 × 10^−4^	*c1*	*−9.6* *×* *10* ^*−3*^	*d1*	*−3.2* *×* *10* ^*−2*^
*a2*	*−3.6* *×* *10* ^*−2*^	b2	4.6 × 10^−3^	*c2*	*−4.6* *×* *10* ^*−3*^		
		b3	1.9 × 10^−2^	*c3*	*−1.1* *×* *10* ^*−2*^		
				*c4*	*−7.4* *×* *10* ^*−3*^		
				c5	4.6 × 10^−2^		
				c6	1.8 × 10^−2^

**Table 2:** Reaction energy barrier of IM5b and ^1^O_2_ in twelve possible reaction pathways unit: Kcal/mol

Energy barrier	a1	a2	c1	c2	c3	c4	d1
∆E	1.01	0.88	20.02	32.94	17.02	18.7	11.86

**Fig. 7:**
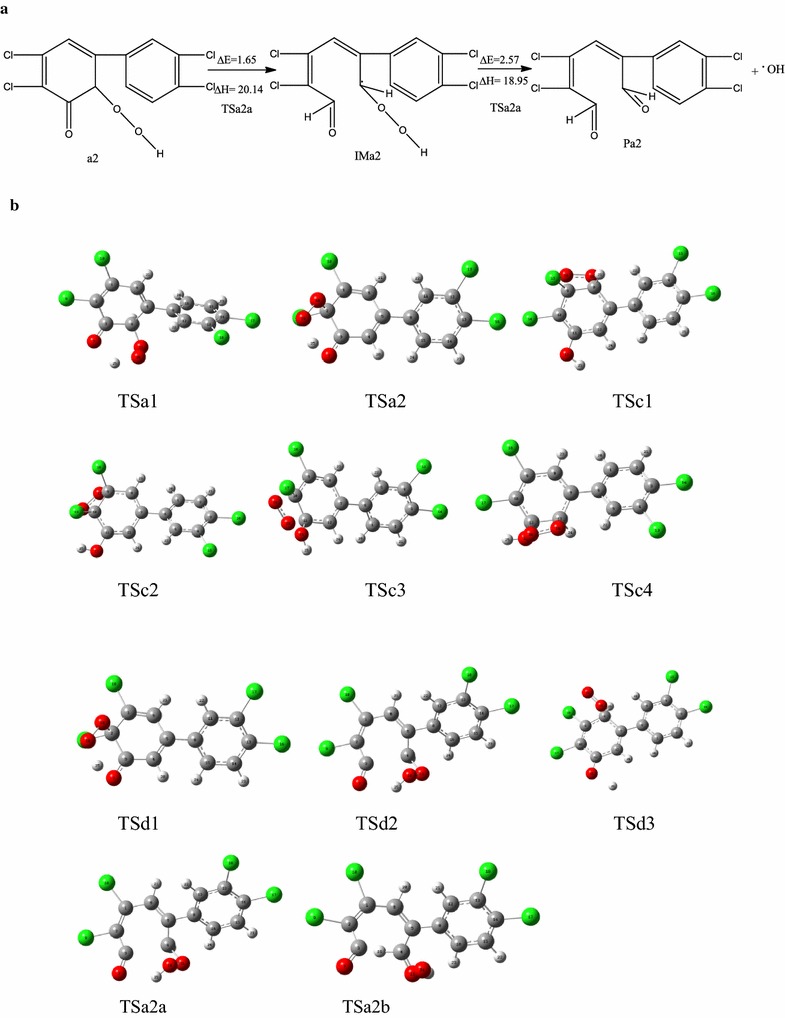
Open loop reaction pathway of intermediate a2 (**a**) and the transition state structure (**b**). [∆E (kcal/mol): energy barrier, ∆H (kcal/mol): reaction heats]

The energy barrier of reaction pathway a2 was the smallest (0.88 kcal/mol), and when the energy barriers of reaction pathways a, c, and d were compared, the energy barrier for pathway a was the smallest. Therefore, IM5b and ^1^O_2_ are more likely to react in a substitution reaction. ^1^O_2_ is preferentially added to the ortho carbon atom, which does not have chlorine substituents. From ·OH, the hydrogen atom migrates to the peroxide group, and the addition of oxygen is carried out at the same time as hydrogen extraction. The pathway for a2 in the atmosphere is shown in Fig. 7a. Because of the lack of stability of ·OOH, the C2–C3 bond was broken into intermediate IMa2 by an open loop reaction with an energy barrier of 1.65 kcal/mol. This reaction is exothermic (20.14 kcal/mol). IMa2 can produce ·OH and aldehyde product Pa2 by monomolecular decomposition with an energy barrier of 2.57 kcal/mol in an exothermic reaction (18.95 kcal/mol). The Optimization structure of transition states as shown in Fig. 7b.

**Fig. 8:**
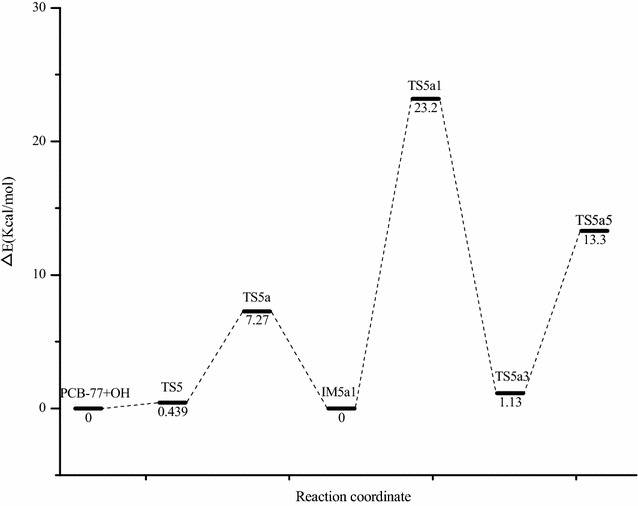
The energy barrier of the reaction path A

After the addition of ·OH, PCB-77 could degrade via pathway A or B. The energy barrier of pathway A is smaller than that of pathway B (Fig. 8). Therefore, PCB-77 preferentially degrades via pathway A in the atmosphere.

### Solvation effect of degradation reaction pathway A

#### The effect of solvation on the reaction energy barrier

From the above study, we can know that the pathway A is the most likely pathway for degradation of PCB-77 in the atmosphere. The solvent has some influence on the reaction and the parameters of the solute, that is, the reaction pathway can be affected by the solvent. We investigated four polar solvents (cyclohexane, benzene, carbon tetrachloride, and chloroform) and seven non-polar solvents (acetone, dichloromethane, ethanol, methanol, acetonitrile, dimethyl sulfoxide, and water). The PCM was used to study the effects of these different solvents on pathway A. Comparison of the reaction energy barriers with the different solvents (Table 3) showed that if the dielectric constant of the solvent was less than five, such as with cyclohexane, carbon tetrachloride, benzene, and chloroform, the energy barrier of was reduced compared to that in the atmosphere. Therefore, PCB-77 is more likely to react with these four solvents than under standard atmospheric conditions, and these solvents could be used to promote this degradation reaction. Among these solvents, benzene could reduce the total reaction energy barrier by 25% compared to that under standard atmospheric conditions. For the solvents with dielectric constants greater than five (dichloromethane, acetone, ethanol, methanol, acetonitrile, dimethyl sulfoxide, and water), the energy barriers were higher than those under standard atmospheric conditions. Therefore, the presence of these solvents would inhibit the reaction. The increase in the dielectric constant would increase interactions between the molecules and change bond lengths [42]. As the dielectric constant increases, it becomes more difficult for PCB-77 to react with the free radicals, and the energy barrier increases because the energy required for bond breaking or formation increases.

**Table 3:** Degradation reaction energy barrier for path A under different solvents unit: Kcal/mol

solvent	*ε*	∆E	∆E1	∆E2	∆E3	∆E4	∆E5
Gas phase	0	0.44	7.27	0	23.2	1.13	13.3
Cyclohexane	2.02	0.24	4.72	0	22.21	0.93	6.72
Carbon tetrachloride	2.23	0.25	4.54	0	22.08	0.87	9.65
Benzene	2.25	0.25	4.66	0	21.43	0.78	7.02
Chloroform	4.9	0.28	5.23	0	22.13	1.02	10.98
Methylene chloride	8.93	0.65	6.27	0	18.32	1.57	14.42
Acetone	20.7	2.38	19.01	0	17.57	1.12	15.42
Ethanol	24.55	0.87	5.45	0	17.44	1.19	16.12
Methyl alcohol	32.63	2.13	25.10	0	17.38	1.01	14.55
Acetonitrile	36.64	1.94	12.68	0	17.31	1.19	17.09
Dimethyl sulfoxide	46.7	2.32	15.03	0	17.31	1.04	14.25
Water	78.39	0.69	8.09	0	17.19	2.82	15.58

#### The effect of solvation on the degradation rate

In this study, the reaction rate constants (*k*) of pathways A and B at 298 K and standard atmospheric pressure were calculated using TST and taking into consideration the tunneling effect. The total reaction rates of pathways A and B were 8.45 × 10^−8^ and 1.15 × 10^−5^ cm^3^/molecule s, respectively. Pathway A is the most likely reaction path, so we then calculated the rate constants for each branch of this pathway, and obtained the Arrhenius equation in the temperature range 238–357 K (Table 4). The reaction rate of PCB-77 with ·OH was 6.67 × 10^−12^ cm^3^/molecule s. Our results cannot be compared with experimental values because of a lack of experimental data. Instead, we compared the rate constants with those of some congeners. A previous study found that the rate constants of PCB-29 (2,4,5-trichlorobiphenyl), PCB-31 (2,4′,5-trichlorobiphenyl), PCB-41 (2,2′,3,5′-tetrachlorobiphenyl) and PCB-47 (2,2′,4,4′-tetrachlorobiphenyl) with ·OH in the atmosphere were 1.3 × 10^−12^, 1.2 × 10^−12^, 0.8 × 10^−12^, and 1.0 × 10^−12^ cm^3^/molecule s at 298 K, respectively [17]. Considering the effect of the degree of chlorine substitution, we think our results agree with the results for these congeners. According to the deduced Arrhenius equation, the reaction rate constant of each pathway increases with increasing temperature. Table 4 shows that the reaction rate for the conversion of IM5a2 to IM5a4 is the fastest among the reactions in this pathway, and this step plays a key role in determining the overall reaction rate. Therefore, if you want to enhance the overall reaction rate, the reaction conditions that affect the pathway should be changed first.

**Table 4:** The degradation rates (k, cm^3^/molecule s) for pathway A at 298 K and standard atmospheric pressure, and the Arrhenius equation at 238–357 K (gas phase)

Reaction path	k_298k_	Arrhenius equation
PCB-77 + ·OH → IM5	6.67 × 10^−12^	5.14 × 10^−13^ exp (−639.12/T)
IM5 + O_2_ → IM5a	2.319 × 10^−10^	4.47 × 10^−12^ exp (−352.60/T)
IM5a1 → IM5a2 + NO_2_	5.54 × 10^−13^	5.79 × 10^−14^ exp (−913.30/T)
IM5a2 → IM5a4	8.43 × 10^−8^	5.13 × 10^−9^ exp (−500.20/T)
IM5a4 + O_2_ → P5a2 + HO_2_	2.33 × 10^−12^	7.25 × 10^−12^ exp (−281.65/T)
		K_total_ = 8.45 × 10^−8^

Different solvents can change the degree of difficulty of a reaction (Table 3). To explore whether the solvent could affect the reaction process and the reaction rates were calculated using different solvents (Table 5). The results suggested the dielectric constants of different solvents would affect the rates of all steps of the reaction path. The reaction rate of the conversion of IM5a2 to IM5a4 increased in cyclohexane, carbon tetrachloride, benzene, dichloromethane, acetone, and ethanol, and this increased the overall reaction rate. Benzene could increase the overall reaction rate by 8% compared to that under standard atmospheric conditions. At the same time, cyclohexane, carbon tetrachloride, and benzene could reduce the energy barrier to improve the reaction rate. Therefore, the reaction conditions can be adjusted to enhance the reaction.

**Table 5:** Degradation Rates (*k*, cm^3^/molecule s) for Pathway A in Different Solvents at 298 K and Standard Atmospheric Pressure

Solvent	*ε*	PCB-77 + ·OH → IM5	IM5 + O_2_ → IM5a	IM5a1 → IM5a2 + NO_2_	IM5a2 → IM5a4	IM5a4 + O_2_ → P5a2 + HO_2_	Ktotal
Gas phase	0	6.67 × 10^−12^	2.319 × 10^−10^	5.54 × 10^−13^	8.43 × 10^−8^	2.33 × 10^−12^	8.45 × 10^−8^
Cyclohexane	2.02	3.15 × 10^−11^	3.91 × 10^−10^	5.23 × 10^−13^	8.98 × 10^−8^	3.14 × 10^−12^	*9.02* *×* *10* ^*−8*^
Carbon tetrachloride	2.23	1.78 × 10^−11^	3.98 × 10^−10^	1.03 × 10^−13^	8.97 × 10^−8^	7.79 × 10^−12^	*9.01* *×* *10* ^*−8*^
Benzene	2.25	1.66 × 10^−11^	4.04 × 10^−10^	4.29 × 10^−13^	9.12 × 10^−8^	5.89 × 10^−12^	*9.16* *×* *10* ^*−8*^
Chloroform	4.9	1.10 × 10^−12^	4.58 × 10^−10^	5.87 × 10^−13^	7.41 × 10^−8^	4.43 × 10^−12^	7.45 × 10^−8^
Methylene chloride	8.93	1.52 × 10^−12^	4.96 × 10^−10^	4.71 × 10^−13^	8.64 × 10^−8^	4.66 × 10^−12^	*8.69* *×* *10* ^*−8*^
Acetone	20.7	2.41 × 10^−12^	3.50 × 10^−11^	1.06 × 10^−13^	8.46 × 10^−8^	4.54 × 10^−12^	*8.46* *×* *10* ^*−8*^
Ethanol	24.6	2.54 × 10^−12^	6.07 × 10^−11^	1.22 × 10^−13^	8.54 × 10^−8^	5.35 × 10^−12^	*8.60* *×* *10* ^*−8*^
Methyl alcohol	32.6	1.59 × 10^−12^	5.49 × 10^−10^	1.46 × 10^−13^	7.08 × 10^−8^	3.15 × 10^−13^	7.08 × 10^−8^
Acetonitrile	36.6	2.62 × 10^−12^	2.81 × 10^−10^	1.45 × 10^−13^	6.88 × 10^−8^	1.30 × 10^−13^	6.88 × 10^−8^
Dimethyl sulfoxide	46.7	1.54 × 10^−12^	5.53 × 10^−10^	1.56 × 10^−12^	6.98 × 10^−8^	1.89 × 10^−12^	8.03 × 10^−8^
Water	78.4	1.91 × 10^−11^	5.58 × 10^−10^	1.73 × 10^−13^	7.42 × 10^−8^	6.54 × 10^−12^	7.47 × 10^−8^

## Conclusions

In this paper, the degradation processes of PCB-77 with ·OH, NO_*x*_, O_2_, and ^1^O_2_ in the atmosphere were evaluated using theoretical calculations. By comparing the energy barriers, the optimal degradation reaction pathway with the minimum energy barrier was selected. The reaction rate of each pathway was calculated by TST, and the effects of different solvents on the reaction pathway were studied based on the solvation effect. We reached the following conclusions:

1. PCB-77 can react with ·OH in ·OH addition and H abstraction reactions in the atmosphere, and ·OH addition is dominant. The ·OH is added to the carbon atoms without substituents. After the addition reaction with ·OH, the reaction can diverge into pathway A or B. The energy barrier of pathway A is lower than that of pathway B, and it is more likely to occur. Most of the pathways in the reaction will produce OH-PCBs, and the OH-PCBs can easily react with singlet oxygen (^1^O_2_). This reaction occurs via one of the three types (a, c, and d) of the four principal types of oxygen addition reactions to aromatic and unsaturated compounds.
2. In the atmosphere, the reaction rates of each step of pathway A increase with increasing temperature, and the final step of the whole reaction process is conversion of IM5a2 to IM5a4.
3. The reaction energy barrier and reaction rate of optimal pathway A are affected by changes in the solvent. Among the investigated solvents, cyclohexane, carbon tetrachloride, and benzene can reduce the reaction energy barrier, and cyclohexane, carbon tetrachloride, benzene, dichloromethane, acetone, and ethanol can improve the reaction rate. The largest reduction in the energy barrier and increase in the reaction rate were obtained with benzene.
